# Larval source reduction with a purpose: Designing and evaluating a household- and school-based intervention in coastal Kenya

**DOI:** 10.1371/journal.pntd.0010199

**Published:** 2022-04-01

**Authors:** Jenna E. Forsyth, Arielle Kempinsky, Helen O. Pitchik, Catharina J. Alberts, Francis M. Mutuku, Lydiah Kibe, Nicole M. Ardoin, A. Desiree LaBeaud

**Affiliations:** 1 Stanford Woods Institute for the Environment, Stanford University, Stanford, California, United States of America; 2 Department of Pediatrics, Stanford University School of Medicine, Stanford, California, United States of America; 3 Division of Epidemiology, School of Public Health, University of California, Berkeley, California, United States of America; 4 Technical University of Mombasa, Mombasa, Kenya; 5 Eastern and Southern Africa Centre of International Parasite Control, Kenya Medical Research Institute, Nairobi, Kenya; Liverpool School of Tropical Medicine, UNITED KINGDOM

## Abstract

**Background:**

Since *Aedes aegypti* mosquitoes preferentially breed in domestic containers, control efforts focus on larval source reduction. Our objectives were to design and test the effectiveness of a source reduction intervention to improve caregiver knowledge and behaviors in coastal Kenya.

**Methodology/Principal findings:**

We conducted a cluster-randomized controlled trial with 261 households from 5 control villages and 259 households from 5 intervention villages. From each household, one child (10–16 years old) and his or her primary caregiver participated in the intervention. We assessed caregiver knowledge and behavior at baseline, as well as 3 and 12 months after the intervention. We assessed household entomological indices at baseline and 12 months after the intervention to avoid seasonal interference. We conducted qualitative interviews with 34 caregivers to understand barriers and facilitators to change. We counted and weighed containers collected by children and parents during a community container clean-up and recycling event. After 12 months, caregiver knowledge about and self-reported behavior related to at least one source reduction technique was more than 50 percentage points higher in the intervention compared to control arm (adjusted risk differences for knowledge: 0.69, 95% CI [0.56 to 0.82], and behavior: 0.58 [0.43 to 0.73]). Respondents stated that other family members’ actions were the primary barriers to proper container management. The number of containers at households did not differ significantly across arms even though children and parents collected 17,200 containers (1 ton of plastics) which were used to planted 4,000 native trees as part of the community event.

**Conclusions/Significance:**

Our study demonstrates that source reduction interventions can be effective if designed with an understanding of the social and entomological context. Further, source reduction is not an individual issue, but rather a social/communal issue, requiring the participation of other household and community members to be sustained.

## Author summary

The disease burden of arboviruses transmitted by *Aedes aegypti* mosquitoes remains high globally. Since these mosquitoes are day-biters, bed nets are not effective at preventing mosquito bites. Additionally, because *Ae*. *aegypti* preferentially breed in domestic containers, control efforts focus on reducing containers for mosquito breeding (source reduction). We developed and evaluated a household- and school-based intervention to improve knowledge and behaviors related to source reduction. We assessed caregiver knowledge and behavior before the intervention and after 3 and 12 months. Subsequently, we conducted qualitative interviews among a sub-set of participants to understand why they did or did not change their behavior. We also assessed mosquito-related indices: the number of breeding containers and immature mosquito abundance before and 12 months after the intervention. We found that those receiving the intervention had more knowledge and self-reported behavior change than those who did not. Nonetheless, the intervention had no effect on mosquito nor containers abundance. In a multi-person household, all household members need to be committed to change, not just the caregiver and child. Our study highlights how mosquito control is not an individual issue, but one that needs the participation of a sufficient majority of household and community members to ensure effectiveness.

## Introduction

*Aedes aegypti* mosquitoes transmit a number of viruses, including dengue and chikungunya [1]. Unlike malaria-transmitting *Anopheles* mosquitoes that bite at night, *Aedes* mosquitoes bite during the day so bed nets are ineffective at preventing disease transmission [2]. Additionally, *Aedes* mosquitoes primarily breed in and around human-made containers and have been shown to be highly resistant to insecticides [3,4]. Therefore, source reduction efforts to physically remove potential breeding habitats represent a critical control strategy [5,6]. The World Health Organization (WHO) recommends an integrated vector management strategy engaging the local health sector and communities in source reduction [7].

For the past two decades, bed net usage across Kenya has increased thanks to numerous well-funded malaria prevention campaigns that focus almost entirely on educating about and distributing free bed nets [8–10]. However, because minimal attention has been paid to non-malaria mosquito-borne diseases, knowledge about source reduction has remained low in coastal Kenya [11]. Although knowledge can be an important antecedent to shifting behaviors, improving content knowledge is insufficient on its own [12]. Content knowledge, combined with procedural or skills-based knowledge, along with support from trusted, respected professional mentors and focused simple behaviors may be more likely to encourage community members to adopt and sustain source reduction behaviors [13]. One study conducted in Greece found that visits by scientific and medical personnel had a greater effect on behavior change than the distribution of printed educational material alone [13].

Past studies of community-based source reduction efforts engaging women and children have successfully reduced mosquito breeding and disease risk [14,15]. Women are frequently engaged in source reduction activities because they are often responsible for household water management in Kenya, as well as other low- and middle-income countries [11]. School-aged children have been considered important agents of change in many public health efforts, including vector control, because they are young, eager to learn, and can propagate interest among family members and communities, possibly increasing adoption [16–18].

A multiple component intervention and randomized-controlled trial conducted in Nicaragua and Mexico involving both women and children resulted in a 29.5% reduction in the risk of dengue virus infection among children and a 52% reduction in pupae per person in households 2 years later [15]. The intervention included peer-to-peer education by school children and community members about multiple ways to accomplish source reduction. Additional activities included demonstrations by community organizations and collective events like street theater, games, and cleanup campaigns.

Another randomized-controlled trial conducted in India demonstrated how targeted source reduction by women’s groups can reduce mosquito abundance [14]. The intervention focused only on covering the most productive containers (cement water tanks) rather than covering or dumping all containers, which may be seen as too labor intensive [14,19]. Women’s groups and community members developed the tailor-made container covers, and schools and other groups shared general educational materials about dengue. The study highlights that engaging women to take on the responsibility of covering the most productive container types was simple and effective, reducing the number of pupae per person by 95% after 10 months.

The aims of this study were to (1) design a caregiver- and child-focused targeted source reduction intervention in coastal Kenya, and (2) evaluate its effectiveness at improving knowledge and source reduction behavior, as well as resulting changes in entomological indices.

## Methods

### Ethics statement

We obtained written informed consent from all study participants (participating caregivers) or their parent/guardian (participating children). The study protocol was reviewed and approved by the ethical review committee at the Kenyatta National Hospital/University of Nairobi and Stanford University.

### Study sites

**Fig 1:**
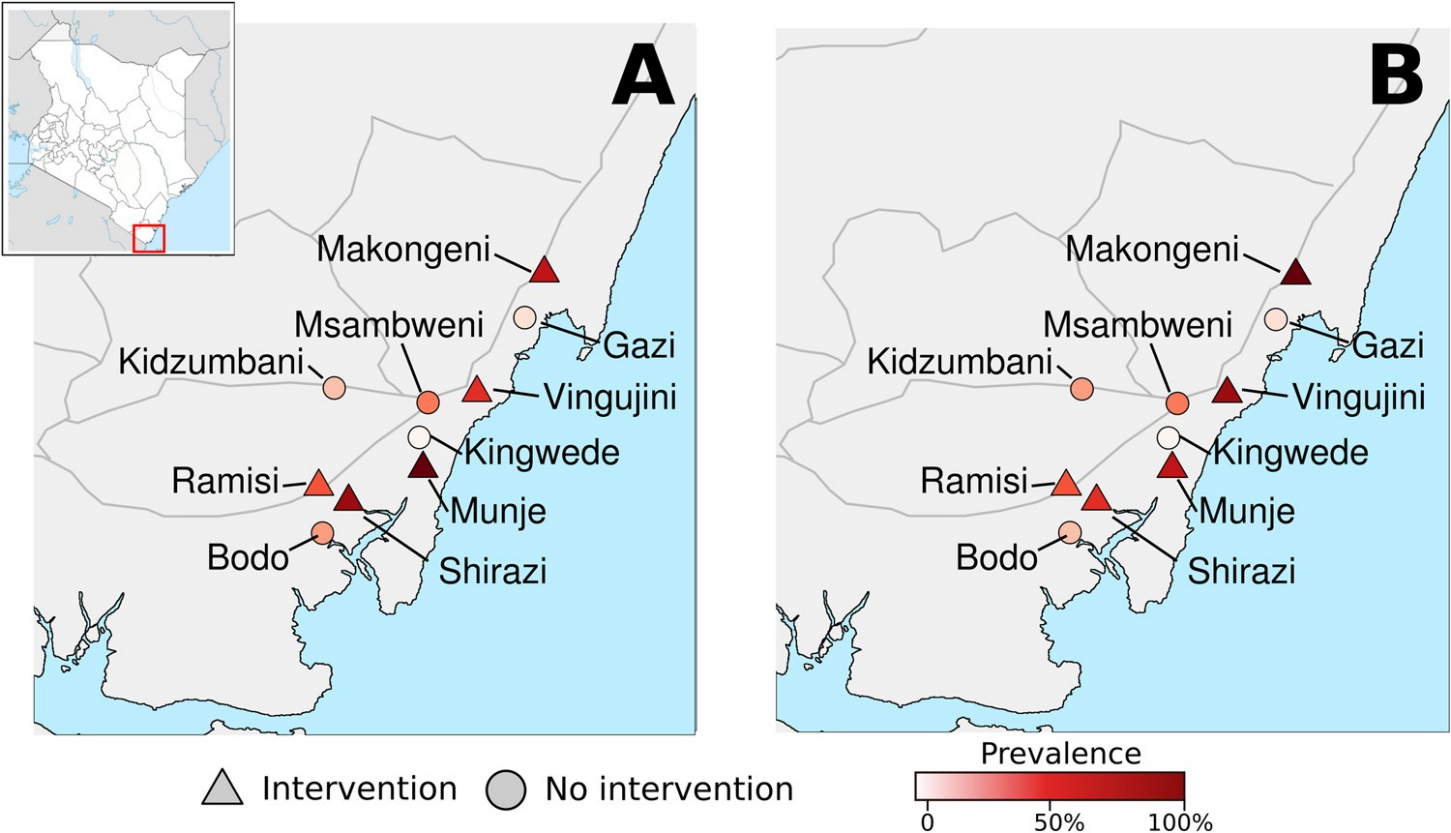
A) Prevalence of knowledge of at least one source reduction behavior to prevent mosquito breeding at 12 months post-intervention in intervention and control villages, and B) prevalence of practicing at least one behavior (e.g., covering containers, removing trash or unused containers, moving containers to a protected enclosure, or poking holes in tires) at 12 months post-intervention in intervention and control villages. Sources: https://data.humdata.org/dataset/ken-administrative-boundaries. The map boundary data was derived from the Humanitarian Data Exchange (HDX) managed by the United Nations Office for the Coordination of Humanitarian Affairs (OCHA) and made available under a Creative Commons Attribution for Intergovernmental Organisations license.

This study was conducted in 10 coastal villages in Kwale County, Kenya, located approximately 60 kilometers south of Mombasa and 50 kilometers north of the Kenya-Tanzania border (4°28′0.0114″S, 39°28′0.12″E) (Fig 1). Seasons are classified based on precipitation levels, which vary considerably throughout the year. The “long dry” season falls between January-March with the lowest precipitation levels. The “long rainy” season falls between April-June with the highest precipitation levels. The “short dry” season falls between July-September, and the “short rainy” season between October-December. Total annual rainfall averages 1,060 mm per year. Annual mean temperatures average 23–34°C with 60–80% relative humidity. For residents, primary domestic water sources vary by season, with rainfall being predominant during the rainy months, and wells and boreholes during the dry months. Residents rely on fishing and subsistence farming for their livelihoods. Islam is the dominant religion. [6]

### Intervention design and piloting

In November 2016, the research team held a 3-day stakeholder workshop to share data about productive container types in these communities [11] and collaboratively design the intervention. The goals of the workshop were to (i) define a logic model for the intervention including resources needed and time involved, (ii) identify source reduction behavioral recommendations for children and caregivers targeting productive mosquito habitats, and (iii) develop messages and activities to generate awareness and motivate the adoption of source-reduction behaviors.

The goal of the curriculum was to focus on as few source reduction behaviors as possible and target only the most productive mosquito breeding habitats. A previous study identified that the most productive mosquito habitats in the region were outdoor containers, particularly those with “no purpose” or those that were left undisturbed for periods of time (e.g., buckets used for laundry) [11]. Intervention activities sought to teach caregivers and children in an interactive and empowering way, giving them the self-efficacy and confidence to practice source reduction on their own.

Between January and March 2017, the team iteratively pilot-tested the intervention in three different villages with a total of 150 school children and 150 caregivers. The piloting was informed by the Trial of Improved Practices Framework [20]. We sought feedback from participants via informal discussions and interviews and aimed to improve and refine the intervention plan after each round of piloting.

### Intervention implementation

We implemented the intervention in a pair-matched cluster-randomized controlled trial where each cluster was a single village. In Kwale County of coastal Kenya, we assessed 25 villages for eligibility (Fig A in S1 Text). Of these, 15 were not eligible for participation based on the following exclusion criteria: (i) located >5km from a main road (n = 7), (ii) no suitable pair village of similar rural/peri-urban status within 5 km (n = 2), or (iii) the village public primary school had recently participated in a research project (n = 6). The 10 remaining villages were then paired up based on proximity to each other and similarities in rural/peri-urban status. The village pairs were then randomized to either the control or intervention group, resulting in 5 intervention and 5 control villages. Eligible participants were caregivers and their children aged 10–16 years attending the public primary school within the village. Within each village, 60 children were randomly selected from the school roster and both the child and caregiver were invited to enroll (see Fig A in S1 Text for trial profile, enrollment, and dropout). The intervention was implemented in the 5 intervention villages across Kwale County of coastal Kenya between May and July 2017. After the final evaluation period in late 2018, the intervention was also implemented in the 5 control villages. Two trained individuals administered the curriculum to caregivers, primarily female heads of households, during a single 1-hour home-based session. The curriculum was also administered to the children of the caregivers at school during 5 consecutive 1-hour interactive after-school lessons. Children and their parents were invited to participate in a container clean-up and plastics recycling event to collect and reuse containers with no immediate purpose. The children brought containers to school to use them to plant seedlings.

### Intervention evaluation—Quantitative analyses

We administered a survey to children and caregivers at baseline, and after 3 and 12 months. At each time point, we assessed participants’ knowledge and behaviors related to source reduction. At baseline we administered a demographic survey to caregivers. At baseline and after 12 months, we administered an entomological survey at the household-level to explore observed behaviors, container counts, and immature mosquito abundance. We estimated differences in outcomes between intervention and control arms at the baseline, 3- and 12-months post-intervention assessments. Primary outcomes were knowledge and self-reported behavior related to source reduction assessed among caregivers assessed at all three time points. Child data were not analyzed due to concerns about validity and completeness. The knowledge question was asked in the following way: “What are the best ways to prevent mosquito breeding?” Similarly, the behavior question was asked: “What do you do to protect yourself from mosquitoes?” Enumerators were trained to ask the question, allowing a pause for the respondent to respond, before asking “anything else?” two times. Enumerators selected behaviors from a list which included both source reduction and general mosquito-borne disease prevention behaviors. The source reduction behaviors of interest were those that were the focus of the intervention content: covering containers, removing trash and unused containers, moving containers out of the rain, and removing or poking holes in tires. Respondents who mentioned at least one of the focal source reduction behaviors were counted and coded as “1” for analyses. Those that did not mention any of the source reduction behaviors were coded as “0.”

Secondary outcomes included standard entomological indices like container index and house index, as well as number of containers per household. These outcome measures were compared twice: at baseline and after 12 months, to avoid seasonal effects.

We estimated risk differences for binary outcomes and mean differences for continuous outcomes adjusting for relevant covariates. Potential covariates included maternal age, maternal education, household size, household assets, and the outcome of interest measured at baseline. For each outcome, covariates were prescreened using a likelihood ratio test, and those with p<0.20 were included in adjusted models. We used the parametric g-formula (R package: riskCommunicator) to estimate marginal differences in outcomes between the intervention and control arm accounting for clustering. This approach follows four steps: (1) fit a regression model, (2) estimate counterfactuals, (3) estimate marginal differences, and (4) construct 95% confidence intervals by bootstrapping resampling with 1,000 replicates. Residuals were normally distributed, and variables did not need to be transformed. Analyses were intention to treat, meaning that they were conducted according to the randomized intervention arm at enrollment, regardless of intervention attendance.

### Intervention evaluation—Qualitative analyses

We conducted qualitative semi-structured interviews 12–15 months after the intervention with 34 purposively selected caregivers to understand the barriers and facilitators to behavior change. Caregivers were selected who demonstrated an increase in knowledge at the 3-month post-intervention assessment in order to focus on the barriers to or facilitators of changing or not changing one’s behavior, assuming the participant understood the benefits. From the subset of caregivers whose knowledge improved, we randomly selected 17 “adopters” who self-reported practicing at least one source reduction behavior and for whom the proportion of covered containers at the 3-month post-intervention assessment either increased or did not change. We randomly selected 17 “non-adopters” who did not self-report practicing source reduction behaviors and for whom the proportion of covered containers either decreased or stayed the same after 3 months. We ensured that adopters and non-adopters were selected proportional to their distribution across the 10 villages (Fig B in S1 Text).

Interviewers used a semi-structured interview and observation guide to explore caregivers’ perceptions and were blinded to adopter status. Interviewers also recorded observations of water containers around the household. Prior to the questions, interviewers first conducted a picture-ranking exercise: the interviewer presented 5 pictures of mosquito-related disease prevention behaviors in random order, naming each as it was presented, and asked the interviewee to place the pictures in order from most to least important. These mosquito-related disease prevention behaviors included sleeping under a bed net, managing water containers (covering, removing, or poking holes), using natural mosquito repellant, burning coconuts and maintaining a clean compound. All interviews were conducted in Kiswahili, audio-recorded, transcribed, and then translated to English. Data analysis followed an inductive and deductive coding process with two independent coders (inter-rater reliability, IRR = 0.83).

## Results

### Intervention design and final content

The stakeholder workshop involved 15 participants including representatives from the Kwale County Ministry of Health, Vector-borne disease control unit, Msambweni Hospital, primary school teachers, behavior change NGOs, and members of a mosquito scout program. After input from stakeholders, we developed the logic model and curriculum. Educational content and behavioral recommendations incorporated theory from the Health Belief Model and other relevant theories (S2 Text and Fig C in S1 Text). Educational content covered the mosquito life cycle with an aim to increase perceived severity and susceptibility of day-time biting mosquitoes and disease. Behavioral content aimed to increase self-efficacy of source reduction by demonstrating different techniques and having caregivers and children practice them. We provided handouts to be kept at home to encourage conversation and cues to action between caregivers and children. Our specific source reduction behavioral recommendations were based on container purpose and the abundance of immature mosquitoes. For children and caregivers, we encouraged them to reduce the number of containers with no immediate purpose. We focused on the collection and re-use of small bottles for seedling and tree planting via a school-based competition. For caregivers, we further encouraged them to focus on covering, turning over, or reducing storage time of containers with a purpose: small containers and buckets for sanitation, buckets/jerry cans for laundry.

Interactive components of the intervention included: mosquito tag to teach about the mosquito life cycle and integrate all concepts into a game for children, artistic images and poetry, and interactive household container mapping whereby a researcher and the participant collectively identified problematic containers at risk of mosquito breeding. The research team covered the content via home visits and school-based lessons, as well as the competition to clean up and re-use no purpose containers.

### Intervention evaluation–quantitative analyses

In total 520 caregivers were enrolled at baseline, with 259 in the intervention arm and 261 in the control arm. At the 3-months post-intervention assessment, 237 in the intervention and 247 in the control arm were followed up with knowledge and behavior questions. While after 12 months, 232 in the intervention and 241 in the control were questioned. Household-based entomological surveys were conducted among 232 in the intervention arm and 248 in the control arm at baseline, and 233 in the intervention arm and 242 in the control arm after 12 months. (Fig A in S1 Text)

Caregivers were predominantly female with a median age of 42 in both intervention and control arms, and for children, the median age was 12 in the control arm and 13 in the intervention arm. The majority of participants were Muslim (99 and 91% of the control and intervention arms), which, for some, meant that extra containers were used to store sanitation water for anal cleansing. Pit toilets were most commonly used (76% and 73% in control and intervention). Concern about mosquitoes was high in the rainy season and low in the dry season among both intervention and control participants (Table 1).

**Table 1:** Baseline characteristics of participants in the control and intervention arms.

Baseline characteristics	Control	Intervention
	(N = 261)^c^	(N = 259)^c^
*Individual*		
Caregiver age (years)^a^	42 (38–47)	42 (38–47)
Caregiver sex (female)	166 (77%)	135 (71%)
Caregiver marital status (married)	164 (76%)	158 (81%)
Child age (years)^**c**^	12 (12–13.5)	13 (12–13)
Child sex (female)	138 (53%)	128 (50%)
*Household*		
Number of people per household	6.0 (2.4)	5.9 (2.1)
Religion (Islam)	213 (99%)	173 (91%)
Main source of water (well/borehole)	124 (57%)	165 (86%)
Boil water before drinking	30 (12%)	40 (15%)
Toilet (pit toilet)	165 (76%)	138 (73%)
Has electricity	71 (33%)	85 (45%)
Owns a television	37 (17%)	50 (27%)
Owns a radio	214 (100%)	184 (96%)
Owns a bicycle	53 (25%)	49 (26%)
*Mosquito-related*		
Use bednets	241 (98%)	232 (98%)
Use natural or synthetic insecticides	39 (16%)	49 (21%)
Clear bushes or grasses	8 (4%)	10 (5%)
Notice mosquitoes on a daily basis^d^		
during the wet season	242 (98%)	232 (98%)
during the dry season	44 (18%)	70 (30%)
Extremely concerned about mosquitoes^d^		
during the wet season	217 (88%)	225 (95%)
during the dry season	35 (14%)	51 (22%)

^a^Median (interquartile range)

^b^Mean (standard deviation)

^c^Percent calculated based on the number of respondents to the given survey (Fig A in S1 Text)

^d^Refers to any type of mosquito, not just day-time biting mosquitoes

Participants in the intervention arm were more likely to both know and self-report practice of at least one source reduction technique at both the 3- and 12-month post-intervention assessments when compared to the control arm (Fig 1 and Table 2). Notably, knowledge about and self-reported behavior of doing at least one source reduction technique was more than 50 percentage points higher in the intervention compared to control arm 12 months after the intervention (adjusted risk difference of knowledge was 0.69, 95% CI [0.56 to 0.82] and behavior was 0.58 [0.43 to 0.73]). After 12 months, covering containers was the most common source reduction behavior reported by participants (reported by 60% of the intervention arm and 10% of the control arm). Although 39% of the intervention arm reported moving containers out of the rain at the 12-month post-intervention assessment, other source reduction behaviors were not adopted as readily. Despite higher self-reported source reduction behavior among the intervention arm, the observed number of covered containers was not significantly different between the arms at any post-intervention time point. (Table 2)

**Table 2:** Knowledge, behavior, and entomological indices at baseline and after 3 and 12 months.

	BASELINE		3 MONTHS			12 MONTHS		
	Control n (%)	Intervention n (%)	Control n (%)	Intervention n (%)	Adjusted Risk Diff. (95% CI)^a^	Control n (%)	Intervention n (%)	Adjusted Risk Diff. (95% CI)
**Knowledge**								
Know at least 1 source reduction technique	27 (11%)	21 (9%)	57 (23%)	142 (65%)	0.44 (0.00, 0.79)	56 (24%)	203 (88%)	0.69 (0.56, 0.82)
Cover containers	25 (10%)	17 (7%)	54 (22%)	126 (58%)	0.36 (-0.08, 0.73)	44 (18%)	171 (74%)	0.64 (0.46, 0.84)
Remove trash and unused containers	3 (1%)	6 (3%)	5 (2%)	64 (29%)	0.34 (0.11, 0.79)	14 (6%)	148 (64%)	0.60 (0.50, 0.76)
Move containers out of rain	0 (0%)	1 (0.4%)	5 (2%)	54 (25%)	0.28 (0.13, 0.55)	15 (6%)	144 (62%)	0.65 (0.52 0.77)
Remove or poke holes in tires	0 (0%)	0 (0%)	0 (0%)	40 (18%)	0.31 (0.08, 0.69)	4 (2%)	26 (11%)	0.10 (0.07, 0.14)
**Behavior**								
*Self-reported*								
Practice at least 1 source reduction technique	3 (1%)	17 (7%)	25 (10%)	101 (46%)	0.41 (0.12, 0.80)	28 (12%)	156 (67%)	0.58 (0.43, 0.73)
Cover containers	3 (1%)	14 (6%)	25 (10%)	88 (40%)	0.33 (0.18, 0.54)	23 (10%)	140 (60%)	0.52 (0.38, 0.64)
Remove trash and unused containers	0 (0%)	4 (2%)	0 (0%)	21 (10%)	0.11 (0.05, 0.16)	3 (1%)	44 (19%)	0.17 (0.13, 0.20)
Move containers out of rain	0 (0%)	0 (0%)	1 (0.4%)	48 (22%)	0.27 (0.21, 0.33)	4 (2%)	90 (39%)	0.45 (0.41, 0.89)
Remove or poke holes in tires	0 (0%)	0 (0%)	0 (0%)	40 (18%)	0.20 (0.20, 0.85)	0 (0%)	5 (2%)	0.02 (0.01, 0.04)
*Observed*								
At least 1 covered container	49 (20%)	34 (15%)	40 (18%)	22 (10%)	-0.07 (-0.16, 0.04)	43 (18%)	37 (16%)	-0.08 (-0.13, -0.01)
Total number of containers^b^	6.3 (3.7)	6.0 (3.5)	4.7 (2.6)	4.6 (2.6)	-0.05 (-0.75, 0.79)	5.2 (3.1)	5.2 (3.2)	-0.05 (-0.98, 0.68
**Entomological Indices**								
Percent of productive containers per house (Container Index)^b^	2.3 (8.4)	2.6 (10.8)	--	--	--	3.9 (16.0)	2.3 (10.4)	-0.01 (-0.04, 0.02)
Houses with at least 1 productive container (House Index)	20 (8%)	19 (8%)	--	--	--	21 (9%)	15 (7%)	0.01 (-0.03, 0.06)

^a^Adjusted for the following baseline characteristics: caregiver age, sex, years of education, number of people in household, drinking water source, toilet type, electricity, bicycle ownership, and baseline level of concern about mosquitoes in the rainy season and reported use of natural or synthetic insecticides.

^b^Mean (SD) and mean differences reported instead of n (%) and risk differences.

The total number of container habitats did not differ significantly between control and intervention arms 12 months after the intervention (adjusted mean difference -0.05, 95% CI [-0.98, 0.68]). Additionally, the container index was similar between the control and intervention arms (adjusted mean difference -0.01, 95% CI [-0.04, 0.02]). (Table 2)

Patterns of container purpose and productivity differed between intervention and control arms at both the baseline and 12-months post-intervention assessments. Overall, containers that were used frequently (e.g., containers for drinking and cooking water) did not support mosquito breeding, likely because the water was emptied before mosquito eggs could hatch. Among the intervention households, containers used for laundry had the most immature mosquitoes at both time points, followed by containers with no purpose and sanitation containers. Among the control households, however, more than 60% of immature mosquitoes were found in containers with no purpose and the rest were found primarily in laundry containers. (Table 3 and Tables A-E in S1 Text)

**Table 3:** Comparison of mosquito habitats by type and purpose at baseline and after 12 months among the control and intervention arms.

**BASELINE**		**CONTROL ARM**								**INTERVENTION ARM**							
		Type (% of habitats)									Type (% of habitats)						
			Bucket (60)	Tire (1)	Jerrycan (24)	Small containers (9)	Drum (3)	Other (3)	Total		Bucket (57)	Tire (1)	Jerrycan (20)	Small containers (11)	Drum (5)	Other (6)	Total
	Purpose (% habitats)	No purpose (4)	31	0	25	6	0	0	62	No purpose (7)	3	9	0	11	0	0	22
		Sanitation (7)	3	0	0	0	0	0	3	Sanitation (8)	29	0	5	0	0	0	34
		Laundry (60)	32	0	0	0	0	0	32	Laundry (49)	13	0	9	5	7	0	34
		Animal water (1)	0	0	0	0	0	0	0	Animal water (3)	0	0	0	0	0	4	4
		Other (28)	2	0	0	0	1	0	3	Other (33)	1	0	0	4	1	0	6
		Total	68	0	25	6	1	0	100	Total	46	9	14	19	8	4	100
**12 MONTHS**			Bucket (49)	Tire (3)	Jerrycan (22)	Small containers (13)	Drum (6)	Other (7)	Total		Bucket (54)	Tire (2)	Jerrycan (18)	Small containers (13)	Drum (7)	Other (6)	Total
	Purpose (% habitats)	No purpose (8)	16	48	0	8	0	0	72	No purpose (8)	21	5	0	13	0	0	39
		Sanitation (7)	0	0	0	0	0	0	0	Sanitation (4)	12	0	0	0	0	0	12
		Laundry (33)	8	0	14	2	4	0	28	Laundry (33)	11	0	27	0	9	0	47
		Animal water (2)	0	0	0	0	0	0	0	Animal water (1)	0	0	0	0	0	2	2
		Other (50)	0	0	0	0	0	0	0	Other (54)	0	0	0	0	0	0	0
		Total	24	48	14	9	4	0	100	Total	44	5	27	13	9	2	100

Percentage of total habitats are shown in parentheses across type and purpose categories. Percent of total immature mosquitoes (both larvae (early and late instars) and pupae) are reported within the cells of the table with shaded color highlighting with green, yellow, orange, and red representing <1%, 1–4.99%, 5–19.99%, and >20% of larval abundance, respectively. Habitat type according to size: 1) small domestic containers, vases, and cooking vessels (<5L), 2) tires, buckets, jerrycans, and basins (10-25L), and 3) drums (>25L). “Other” purpose included bathing, drinking cooking, and multiple functions. Total numbers of immature mosquitoes and habitats varied by arm and time point. For total immature mosquitoes, baseline control n = 716 and 12 months n = 642; baseline intervention n = 791 and 12 months n = 419. For habitat numbers, baseline control n = 1,009 and 12 months n = 823; baseline intervention n = 705 and 12 months n = 816.

During recycling events, children collected 17,200 containers (1 ton of plastic) and planted 4,000 native trees. 3,754 containers were deemed unusable and were buried in a subterranean containment given the lack of proper waste management and disposal in the county. 1,000 containers were unusable but did not fit in the burial pit. At endline, 81% of intervention caregivers mentioned that their children discussed the intervention with them (Table F in S1 Text).

### Intervention evaluation–qualitative analyses

The post-intervention qualitative interviews revealed that all but one respondent intended to cover containers (n = 33). Although adopters were two times more likely than non-adopters to mention the intent of moving containers to a protected enclosure (n = 9 vs. 4), the rates of other source reduction behaviors were similar among adopters and non-adopters. Overall, adopters reported more benefits from the intervention than non-adopters (e.g., they were 23% more likely to mention having cleaner compounds). (Table G in S1 Text) Notably, during the picture ranking exercise, seven adopters ranked proper container management second, after having a clean compound, while seven non-adopters ranked it third out of five (Fig D in S1 Text).

The most frequently mentioned barrier to behavior change was interference from others, especially children who would use containers as toys (n = 17 affected by children’s behavior, n = 7 affected by the behavior of other adults). Additional barriers included the difficulty managing a large number of containers on top of other household duties (n = 18), followed by losing container covers (n = 13). Even adopters relayed these concerns, adding that they would cover additional containers if they had lids. The two primary facilitators to behavior change were having an ongoing concern about hygiene and disease prevention (n = 28) (e.g., dedication to keeping compound clean, using bednets to prevent malaria, etc.), and having containers filled with water used for consumption (n = 28) (e.g., drinking water containers were covered to keep the water potable). (Table 4)

**Table 4:** Barriers to and facilitators of proper container management described by adopters and non-adopters during follow-up semi-structured in-depth interviews. The n and % refer to the number of respondents who mentioned a sub-theme.

Theme	Sub-theme	Adopters n (%)	Non-adopters n (%)	Notes and Quotes
**Barriers**	Behavior of Others	13 (76%)	11 (69%)	Children creating water sources for mosquitoes by leaving plastic containers and tires behind that they used as toys, as well as neighbors and other adults leaving open containers behind consciously or unconsciously. **"Yes, there are challenges…like when I clean my compound and my neighbors do not, mosquitos will fly from their place to my house."** (A2007)
	Number of Containers	11 (65%)	7 (41%)	Too much trash and unused containers to deal with.
	Lack of Resources	7 (41%)	6 (35%)	When asked why uncovered containers are numerous. . . **"It is because they don’t have covers. Those containers outside, I cover them but the children remove the covers and throw them away."** (A1016)
	Weather (Rain)	4 (24%)	4 (24%)	Intentional use of buckets and basins to store rain water, as well as unintentional rainwater accumulation in no purpose containers.
	Lack of Time	3 (18%)	5 (29%)	Numerous competing commitments during the day.
**Facilitators**	Hygiene/Disease Concern	15 (88%)	13 (76%)	Use of bed nets, and dedication to maintaining compound cleanliness and order.
	Purpose of water use is for consumption	16 (94%)	12 (71%)	Containers holding drinking and cooking water "needed" to be covered.
	Respect	10 (59%)	11 (65%)	Appreciated the time spent at the house and valued the study team providing recommendations
	Gender Norms	2 (12%)	3 (18%)	Responsibility as a woman to maintain the compound. **"It is not difficult covering water containers because that is the role of a woman."** (A2004)

## Discussion

This study combined qualitative and quantitative methods to develop and evaluate a multiple component source reduction intervention consisting of an interactive curriculum and a community container clean-up and reuse event. By conducting both qualitative and quantitative evaluations of the intervention, we were able to identify important considerations for subsequent efforts to reduce the burden of mosquito-borne disease from *Aedes* mosquitoes. Ultimately, the infestation of container-breeding mosquitoes in communities is a collective action problem affecting both human and environmental health and thus requires multi-faceted solutions.

We observed a significant increase in knowledge and self-reported behavior among caregivers in the intervention arm compared to the control arm. This may be due, in part, to the pedagogical style used by the study team, as well as the activity-driven curriculum with built-in repetition to foster learning and promote mastery of concepts [21–24]. This may also be due to the alignment of certain behavioral recommendations with current hygiene norms. Covering water containers was the most reported source reduction behavior because respondents wanted to keep water clean, especially water used for drinking and cooking. However, because so few immature mosquitoes were found in containers used for drinking and cooking, covering these containers was not a highly effective source reduction behavior. The intervention encouraged participants to remove or cover containers with the most immature mosquitoes (e.g., containers with no purpose or those for laundry and sanitation), but these recommendations were not as widely adopted by participants.

Source reduction was a novel concept among this community as a way to mitigate mosquito-borne disease. Therefore, continued effort would be needed to encourage source reduction behaviors aside from container covering as they are not commonplace nor particularly aligned with pre-existing concerns. Respondents continued to report that bed nets were the most valued approach for preventing mosquito-borne disease despite the intervention’s focus on source reduction behaviors. This is not surprising since campaigns to promote bed net usage have been ongoing in Kenya and other countries in Sub-Saharan Africa since the 1950s [25], whereas this was the first known effort to promote source reduction in Kwale county. A recent study conducted in the study region also determined that locals perceive day-time biting *Ae*. *aegypti* mosquitos as less of a health risk than malaria-causing *Anopheles* mosquitoes; that their bite would be less likely to get you sick [11]. Moreover, doctors in the region tend to diagnose any febrile illness as malaria when it might be another arboviral disease like dengue or chikungunya, so the perceived threat of malaria being higher is perpetuated in the health system [26].

Our study highlighted how the management of no purpose containers and waste should include other household members, as well as neighbors and the broader community. For example, since all family members interact with containers in the household, someone could uncover a container after a caregiver had intentionally covered the container. The same is true for other behaviors, like moving containers to a protected enclosure, or turning over containers when they are not in use. Children preferred keeping tires at the home instead of disposing them so they could be used as toys, which was problematic since tires were some of the most productive container types. Caregivers were an appropriate focal point for the intervention due to their primary responsibilities related to water collection and storage, but adhering to new container management requires commitment from entire households.

Coordinated clean-ups that motivate community members to remove no purpose containers and other breeding sites from the environment are more likely to be impactful than expecting individuals or households to change daily habits associated with container management. As some participants noted, a household’s commitment to source reduction may not confer a demonstrable benefit in terms of mosquito-related risk reduction if a neighboring household has numerous no purpose containers that serve as breeding grounds for *Ae*. *aegypti*.

The container clean-up and recycling event as part of the intervention brought together parents, children, and teachers and reinforced a sense of community. Nearly one ton of plastics were collected during the clean-up event. Nonetheless, the lack of observed change in the number of household containers suggests that either children focused on collecting plastics in their communities and not their immediate households or that plastic containers accumulate extremely quickly around a child’s home that the effort would need to be repeated frequently in order to show impact. Moreover, some containers truly had no purpose (essentially trash), while others had no immediate purpose but were being kept in case of need. Either category poses a risk because rain can transform any unattended uncovered container into a breeding habitat.

Without a system of managing the volume of solid waste generated in Kenya, individual and community clean-up efforts would be inadequate to address the problem of no purpose containers. Approaches are needed to reduce, reuse, recycle, and safely dispose of plastic bottles, containers, and tires. Our intervention offered some use for no purpose containers by planting seedlings in them, but numerous containers were deemed unusable. We opted to bury these unusable containers since there was no other option to recycle or dispose of them. If left in communities, the most common approach to eliminating accumulated trash is to burn it: in sub-Saharan Africa, more than 75% of waste is burned [27]. Burning trash is dangerous for the human health as well as the environment [28–30] as it releases toxic chemicals into the air and pollutes the environment while increasing greenhouse gasses contributing to climate change [27].

Large amounts of plastic are being produced and accumulating in the absence of a circular economy to recoup these materials. Glass and other materials are valuable enough to incentivize their recycling in Kenya, but not lower quality plastics [31–33]. Efforts to generate profitable uses for single use plastics, or policies to reduce the production and sale of these plastic bottles and other containers could be effective. The Kenyan government has already demonstrated an ability and commitment to reducing plastic waste by imposing and enforcing the world’s strictest plastic bag policy [34,35].

Ultimately, many levers for change exist, and individuals cannot be expected to reduce plastics on their own. The combination of individual and household-level behavior change, coordinated community clean-ups, structural changes incentivizing reuse and recycling, and targeted policies to restrict plastic production could all accelerate progress towards source reduction to prevent mosquito breeding.

The intervention had notable strengths, as documented by improvements in knowledge and behaviors, and participation in the clean-up event. However, there were also limitations. One limitation related to our inability to assess changes in knowledge and behavior among school children. Due to the many demands placed on schools and their rigid schedules for learning, we were unable to get permission to visit the schools multiple times to conduct follow-up assessments. Another limitation was that we were unable to determine how effective the intervention was at reducing mosquito abundance and arboviral disease. The abundance of immature mosquitoes in the study site was low. Conducting a similar intervention in a nearby urban community where population density, mosquito abundance, and disease incidence are higher would be worthwhile and possibly allow for these outcomes to be powered for analyses.

## Conclusions

Through the course of this intervention, we documented changes in knowledge and self-reported behavior, with a focus on covering containers for drinking and cooking. Behavior change around removing and covering the highly productive laundry, sanitation or “no purpose” containers was not as effective. Community clean-ups were a successful way to engage children and incentivize the collection of no purpose containers. Household water container management was not a high priority among participants, so behavior change was most evident when source reduction behaviors aligned with other hygiene-related behavior. Source reduction is not an individual issue, but a household and community issue, so interventions need to engage entire households and communities for long-term change. Structural changes such as improving solid waste collection, recycling, and disposal services are needed to reduce the volume of plastic waste and mosquito breeding in the region.

## Supporting information

S1 Text Supplementary figures and tables. (DOCX)

S2 Text Intervention curriculum. (DOCX)
