# Supplementary material for: Larval source reduction with a purpose: Designing and evaluating a household- and school-based intervention in coastal Kenya
Source: PLoS Negl Trop Dis. 2022 Apr 1;16(4):e0010199. doi: 10.1371/journal.pntd.0010199 (PMC9007363; doi:10.1371/journal.pntd.0010199)
Supplement: S1 Text — (DOCX) [file pntd.0010199.s001.docx]

SUPPORTING INFORMATION

Full title: **Larval source reduction with a purpose: designing and evaluating a household- and school-based intervention in coastal Kenya**

Short title: Household- and school-based larval source reduction intervention in coastal Kenya

Jenna E. Forsyth,^1*^ Arielle Kempinsky,^2^ Helen O. Pitchik,^3^ Catharina Alberts,^2^ Francis M. Mutuku,^4^ Lydiah Kibe,^5^ Nicole Ardoin,^1^ A. Desiree LaBeaud^2^

^1^Stanford Woods Institute for the Environment, Stanford University, Stanford, California, U.S.A.

^2^Stanford University School of Medicine, Stanford, California, U.S.A.

^3^University of California, Berkeley, U.S.A.

^4^Mswambweni District Hospital’s Vector-Borne Disease Unit, Mswambweni, Kenya

^5^Centre for Geographic Medicine Research Coast, Kenya Medical Research Institute, Kilifi, Kenya

***Corresponding author:** Jenna E. Forsyth, jforsyth@stanford.edu

**Running title:** Source reduction intervention improves knowledge and behavior in coastal Kenya

**FIGURES**

Figure A. Flow diagram of the trial profile, including eligibility, enrollment, and participation in each data collection step.

Figure B**.** Flow diagram illustrating the selection process for 17 adopters and 17 non-adopters to be interviewed about the intervention during July-August 2018 after the 12 month follow-up surveys.

Figure C. The mosquito detective badge showing the different types of most productive containers.

Figure D. Importance rankings of “container management” among adopters and non-adopters. Respondents were asked to rank the following five mosquito-related disease prevention behaviors in order of importance from most (1) to least (5) important: container management (source reduction), sleeping under a bed net, using natural mosquito repellant, burning coconuts and maintaining a clean compound.

**TABLES**

Table A. Knowledge and behavior survey responses: container management practices at baseline, 3, and 12 months.

Table B. Knowledge and behavior survey responses: know and practice source reduction techniques at baseline, 3, and 12 months.

Table C. Entomologic survey responses: container purpose at baseline and 12 months.

Table D. Entomologic survey responses: habitat type at baseline and 12 months.

Table E. Entomologic survey responses: water source at baseline and 12 months.

Table F. Knowledge and behavior survey responses: communication at baseline, 3, and 12 months.

Table G. Adopter and non-adopter behavioral intention and perceived benefits of the intervention.

**FIGURES**

Figure A. Flow diagram of the trial profile, including eligibility, enrollment, and participation in each data collection step.

K=knowledge and behavior survey responders; E=entomological survey responders

Figure B**.** Flow diagram illustrating the selection process for 17 adopters and 17 non-adopters to be interviewed about the intervention during July-August 2018 after the 12 month follow-up surveys.

Figure C. The mosquito detective badge showing the different types of most productive containers.


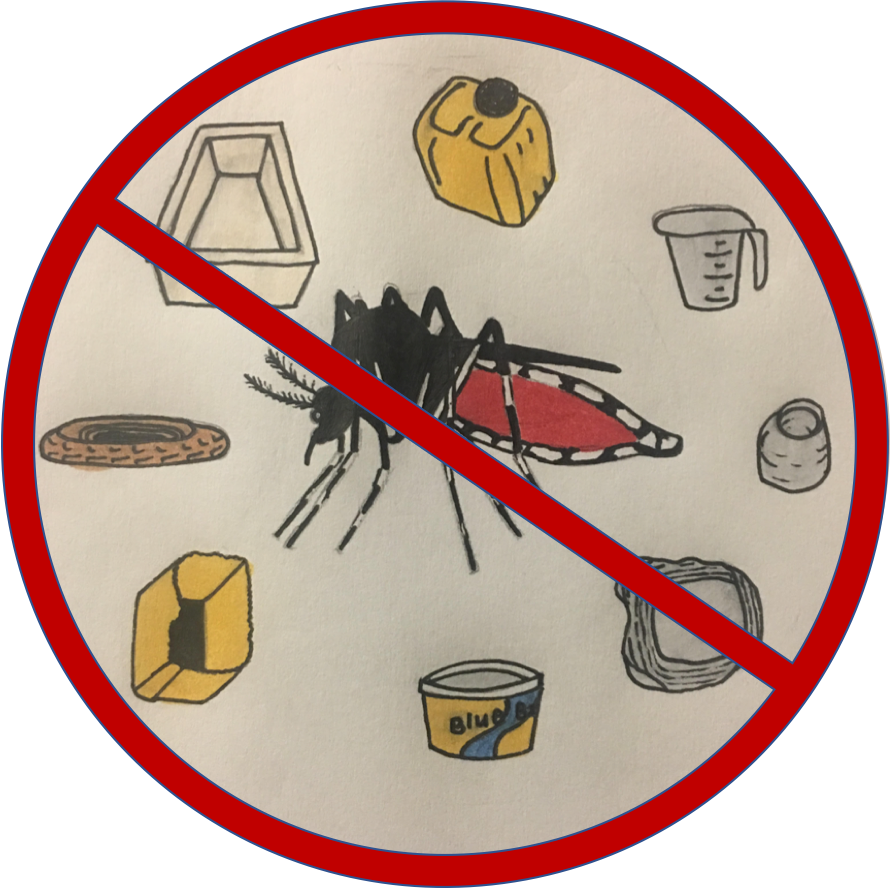


Figure D. Importance rankings of “container management” among adopters and non-adopters. Respondents were asked to rank the following five mosquito-related disease prevention behaviors in order of importance from most (1) to least (5): container management (source reduction), sleeping under a bed net, using natural mosquito repellant, burning coconuts and maintaining a clean compound.

**TABLES**

Table A. Knowledge and behavior survey responses: container management practices at baseline, 3, and 12 months.

|  | BASELINE | | 3 MONTHS | | 12 MONTHS | |
| --- | --- | --- | --- | --- | --- | --- |
|  | CONTROL | INTERVENTION | CONTROL | INTERVENTION | CONTROL | INTERVENTION |
|  | n=247 | n=237 | n=250 | n=219 | n=241 | n=232 |
| **Knowledge & Behavior** |  |  |  |  |  |  |
| Cover containers | 2 (1%) | 7 (3%) | 23 (9%) | 83 (38%) | 19 (8%) | 114 (49%) |
| Remove trash and unused containers | 0 (0%) | 2 (1%) | 0 (0%) | 20 (9%) | 2 (1%) | 42 (1%) |
| Move containers out of rain | 0 (0%) | 0 (0%) | 1 (0%) | 33 (15%) | 3 (1%) | 70 (30%) |
| Remove or poke holes in tires | 0 (0%) | 0 (0%) | 0 (0%) | 23 (11%) | 0 (0%) | 2 (1%) |

Table B. Knowledge and behavior survey responses: know and practice source reduction techniques at baseline, 3, and 12 months.

|  | BASELINE | | 3 MONTHS | | 12 MONTHS | |
| --- | --- | --- | --- | --- | --- | --- |
|  | CONTROL | INTERVENTION | CONTROL | INTERVENTION | CONTROL | INTERVENTION |
| **Knowledge & Behavior** |  |  |  |  |  |  |
| Know at least 2 source reduction techniques | 1 (0.4%) | 3 (1%) | 6 (2%) | 79 (31%) | 18 (7%) | 167 (64%) |
| Practice at least 2 source reduction techniques | 0 (0%) | 1 (0.4%) | 1 (0.4%) | 65 (25%) | 2 (0.8) | 87 (34%) |

Table C. Entomologic survey responses: container purpose at baseline and 12 months.

| **CONTROL** | BASELINE | | |  | ENDLINE | | |  |
| --- | --- | --- | --- | --- | --- | --- | --- | --- |
| Container purpose | No. of containers | No. of positive containers | No. of immature mosquitoes |  | No. of containers | No. of positive containers | No. of immature mosquitoes |  |
| Bathing | 50 | 1 | 12 |  | 28 | 0 | 0 |  |
| Drinking | 10 | 0 | 0 |  | 4 | 0 | 0 |  |
| Cooking | 23 | 1 | 10 |  | 12 | 0 | 0 |  |
| Animals | 9 | 0 | 0 |  | 15 | 0 | 0 |  |
| No purpose | 58 | 6 | 442 |  | 103 | 16 | 460 |  |
| Laundry | 603 | 16 | 231 |  | 275 | 11 | 182 |  |
| Sanitation | 68 | 2 | 21 |  | 56 | 0 | 0 |  |
| Other and multiple functions | 188 | 0 | 0 |  |  | 3 | 0 |  |
|  | **1009** | **26** | **716** |  | **823** | **30** | **642** |  |
| **INTERVENTION** | |  |  |  |  |  |  |  |
| Container purpose | No. of containers | No. of positive containers | No. of immature mosquitoes |  | No. of containers | No. of positive containers | No. of immature mosquitoes | |
| Bathing | 52 | 1 | 6 |  | 17 | 0 | 0 |  |
| Drinking | 10 | 0 | 0 |  | 4 | 0 | 0 |  |
| Cooking | 12 | 0 | 0 |  | 10 | 0 | 0 |  |
| Animals | 18 | 1 | 32 |  | 7 | 1 | 10 |  |
| No purpose | 74 | 7 | 176 |  | 98 | 7 | 163 |  |
| Laundry | 342 | 8 | 267 |  | 269 | 6 | 197 |  |
| Sanitation | 55 | 7 | 266 |  | 34 | 3 | 49 |  |
| Other and multiple functions | 142 | 3 | 44 |  | 377 | 0 | 0 |  |
|  | **705** | **27** | **791** |  | **816** | **17** | **419** |  |

Table D. Entomologic survey responses: habitat type at baseline and 12 months.

| **CONTROL** | BASELINE | | | | |  | ENDLINE | | | | |
| --- | --- | --- | --- | --- | --- | --- | --- | --- | --- | --- | --- |
| Habitat type | No. of containers | % filled with rainwater | No. of positive containers | % of positive containers filled with rainwater | No. immature mosquitoes |  | No. of containers | % filled with rainwater | No. of positive containers | % of positive containers filled with rainwater | No. immature mosquitoes |
| Buckets | 604 | 372 | 18 | 15 | 485 |  | 406 | 168 | 10 | 9 | 156 |
| Jerrycan | 244 | 137 | 5 | 3 | 181 |  | 183 | 59 | 6 | 3 | 93 |
| Small container | 87 | 46 | 2 | 1 | 40 |  | 105 | 57 | 4 | 4 | 60 |
| Drum | 26 | 19 | 1 | 0 | 10 |  | 45 | 34 | 2 | 2 | 27 |
| Tire | 14 | 14 | 0 | 0 | 0 |  | 26 | 26 | 8 | 8 | 306 |
| Other | 34 | 10 | 0 | 0 | 0 |  | 58 | 9 | 0 | 0 | 0 |
|  | **1009** | **598** | **26** | **19** | **716** |  | **823** | **353** | **30** | **26** | **642** |
| **INTERVENTION** |  |  |  |  |  |  |  |  |  |  |  |
| Habitat type | No. of containers | % filled with rainwater | No. of positive containers | % of positive containers filled with rainwater | No. immature mosquitoes |  | No. of containers | % filled with rainwater | No. of positive containers | % of positive containers filled with rainwater | No. immature mosquitoes |
| Buckets | 402 | 142.0 | 11 | 4.0 | 361 |  | 441 | 310 | 10 | 7 | 183 |
| Jerrycan | 143 | 32.0 | 4 | 3.0 | 109 |  | 146 | 97 | 1 | 1 | 115 |
| Small container | 74 | 35.0 | 5 | 4.0 | 151 |  | 103 | 81 | 2 | 2 | 55 |
| Drum | 35 | 20.0 | 3 | 3.0 | 67 |  | 55 | 50 | 2 | 2 | 37 |
| Tire | 10 | 10.0 | 2 | 2.0 | 68 |  | 17 | 17 | 1 | 1 | 19 |
| Other | 41 | 8.0 | 2 | 1.0 | 35 |  | 54 | 15 | 1 | 1 | 10 |
|  | **705** | **247.0** | **27** | **17** | **791** |  | **816** | **570** | **17** | **14** | **419** |

Table E. Entomologic survey responses: water source at baseline and 12 months.

| **CONTROL** | BASELINE | | |  | ENDLINE | | |
| --- | --- | --- | --- | --- | --- | --- | --- |
| Water source | No. of containers | No. of positive containers | No. of immature mosquitoes |  | No. of containers | No. of positive containers | No. of immature mosquitoes |
| Rain | 598 | 19 | 547 |  | 353 | 26 | 541 |
| Borehole | 61 | 1 | 35 |  | 123 | 0 | 0 |
| Well | 125 | 4 | 102 |  | 81 | 0 | 0 |
| Public tap | 206 | 2 | 32 |  | 212 | 4 | 101 |
| Pond/Stream | 0 | 0 | 0 |  | 14 | 0 | 0 |
|  | **1009 (19=none)** | **26** | **716** |  | **823 (40=none)** | **30** | **642** |
| **INTERVENTION** | |  |  |  |  |  |  |
| Water source | No. of containers | No. of positive containers | No. of immature mosquitoes |  | No. of containers | No. of positive containers | No. of immature mosquitoes |
| Rain | 247 | 17 | 443 |  | 570 | 14 | 370 |
| Borehole | 122 | 3 | 81 |  | 65 | 3 | 49 |
| Well | 269 | 7 | 257 |  | 92 | 0 | 0 |
| Public tap | 45 | 0 | 0 |  | 50 | 0 | 0 |
| Pond/Stream | 0 | 0 | 0 |  | 2 | 0 | 0 |
|  | **705 (22=none)** | **27** |  |  | **816 (37=none)** | **17** | **419** |

Table F. Knowledge and behavior survey responses: communication at baseline, 3, and 12 months.

|  | BASELINE | | 3 MONTHS | | 12 MONTHS | |
| --- | --- | --- | --- | --- | --- | --- |
|  | CONTROL | INTERVENTION | CONTROL | INTERVENTION | CONTROL | INTERVENTION |
| **Communication** |  |  |  |  |  |  |
| Parents and children discuss school logistics and fees | 125 (48)% | 62 (24)% | 63 (24)% | 96 (37)% | 149 (57% | 155 (60)% |
| Parents and children discuss school curricular content | 87 (33)% | 80 (30)% | 71 (27)% | 89 (34)% | 159 (61)% | 132 (51)% |
| Parents and children discuss school intervention | 0 (0)% | 0 (0%) | 14 (5)% | 197 (76)% | 6 (2)% | 209 (81)% |

Table G. Adopter and non-adopter behavioral intention and perceived benefits of the intervention.

| **Theme** | **Sub-theme** | **Adopters** | **Non-adopters** | **Quotes** | **Notes/Observations** |
| --- | --- | --- | --- | --- | --- |
| **Behavioral intention** | Cover container | 17 (100%) | 16 (94%) | Why do you cover your water containers? **To maintain the cleanliness of the water, while preventing insect-like mosquitos from entering and breeding inside** (A3045) | Jerry can openings are blocked with cut water bottles or socks when fitted covers were not available |
|  | Move container to enclosure | 9 (53%) | 4 (24%) |  | Water containers stored under awnings |
|  | Dump out water/Flip over container | 8 (47%) | 8 (47%) | **Turning upside down unused containers destroys mosquito breeding sites, reducing the number of mosquitos** (A2030) | Pots on sticks upside down, plastic containers hanging upside down in tree |
|  | Reduce number/Remove no purpose containers | 6 (35%) | 5 (29%) | **When I get rid of the un-needed containers, mosquitos cannot breed inside and therefore, there will be fewer in number** (A2001) | Overall cleaning the compound was mentioned as a related overarching category |
| **Benefits of the intervention** | Decreased sickness | 10 (59%) | 11 (65%) | What changed in your house after the intervention? **The cleanliness has greatly improved** How do these changes impact your daily life? **Nowadays we do not get sick easily, like this year we have been sick once** (A2001) | Noted decrease is sickness on the compound since intervention |
|  | Cleaner compound | 16 (94%) | 12 (71%) | What is the difference between now and before [the intervention]? **There is not even coconut shells -- when my kids come from school, they throw them away and tell me that they were told to clean the compound** (A3015) | Compounds with absolutely no trash and/or no purpose containers lying around. |
|  | Reduced number of mosquitos | 5 (29%) | 3 (18%) | What did you change around your home after the school program? **When my children come home from school, they always remind me to cover our water containers** (A3023) | Less mosquitos noted to be flying around compound |
|  | Increased knowledge | 11 (65%) | 6 (35%) | Did you learn anything new from the intervention?  **I learned that the mosquitos love to breed in the standing water inside of bottles** (A5004) | Trees planted in repurposed water bottles |

### 
