# Supplementary material for: Larval source reduction with a purpose: Designing and evaluating a household- and school-based intervention in coastal Kenya
Source: PLoS Negl Trop Dis. 2022 Apr 1;16(4):e0010199. doi: 10.1371/journal.pntd.0010199 (PMC9007363; doi:10.1371/journal.pntd.0010199)
Supplement: S2 Text — (DOCX) [file pntd.0010199.s002.docx]

SUPPORTING INFORMATION

Full title: **Larval source reduction with a purpose: designing and evaluating a household- and school-based intervention in coastal Kenya**

Short title: Household- and school-based larval source reduction intervention in coastal Kenya

Jenna E. Forsyth,^1*^ Arielle Kempinsky,^2^ Helen O. Pitchik,^3^ Catharina Alberts,^2^ Francis M. Mutuku,^4^ Lydiah Kibe,^5^ Nicole Ardoin,^1^ A. Desiree LaBeaud^2^

^1^Stanford Woods Institute for the Environment, Stanford University, Stanford, California, U.S.A.

^2^Stanford University School of Medicine, Stanford, California, U.S.A.

^3^University of California, Berkeley, U.S.A.

^4^Mswambweni District Hospital’s Vector-Borne Disease Unit, Mswambweni, Kenya

^5^Centre for Geographic Medicine Research Coast, Kenya Medical Research Institute, Kilifi, Kenya

***Corresponding author:** Jenna E. Forsyth, jforsyth@stanford.edu

**Running title:** Source reduction intervention improves knowledge and behavior in coastal Kenya

### **Aedes Intervention Curriculum**

**ROLES and RESPONSIBILITIES**

*ROLE OF TEACHERS IN CLASS SESSIONS*

Collect pre- and post-evaluation test checking that we receive one from each student and that each student followed instructions

Organize classroom and plan session venue

Re-arrange the seating arrangements of the students each day (mixing top and poor performing students)

Prepare class attendance and take roll call on daily basis

Help in taking around containers with larvae pupa and adult mosquitoes each day

Help in recap sessions

Collect homework on days 2, 4, and 5

Record on the roll call sheet which students we have received homework from

Ensure that the student wrote their name and followed instructions

Make sure each student has a notebook and pen to take notes with on a daily basis

Maintain class focus and address and behavioral problems or troublesome students on a daily basis

*ROLE OF KAMONI and MWASHEE IN CLASS SESSIONS*

Teach

Record which students participate in mosquito tag

Grade homework

Verify that pre- and post- evaluation tests were filled out correctly

Verify that homeworks were filled out correctly

**TOPICS and TIMELINE**

*Day 0, Student Pre-Test*

Materials needed

Pre-tests

Description

Students take a written pre-test to understand baseline knowledge and behaviors.

This occurs the Friday before the intervention starts (which is on Monday).

Teachers will help administer the pre-test

*Day 1, Topic 1:* Mosquito life cycle

Materials needed

Live larvae, pupae and adults

Small slips of paper for students to write what they see in the container with pupae/larvae

Mosquito life cycle drawings

Mosquito tag rubber bands

Blank sheets of paper for homework #1

What students should know

Four stages in the mosquito lifecycle and which are in water versus air: eggs (water), larvae (water), pupae (water), adult (air).

**Implication:** Water matters for lifecycle, females bite for blood to lay eggs –larvae have potential for harm (water isn’t poison it’s just an indication that mosquitoes can emerge).

Duration of the mosquito lifecycle – how much time from egg to adult –no need to know days of each of the 4 stages. The life cycle is 7-14 days.

**Implication:** Must cover or dump containers NOW (before 7 days has passed) to prevent breeding. Drinking water is used daily so is unlikely to breed mosquitoes. Other water that sits for days is more likely.

Assignments and activities

A student familiarization session to take place just before starting up. The first thing to do should be passing around containers with mosquito larva, pupa, and adults as well as eggs. After each student examines the larva, pupa, and adults, ask the children to write down what they have seen in the containers. (20 minutes)

Bring in each of the four life cycle stages to let the students see them (egg, larvae, pupae, and adult). Keep these in a safe place at the school for the whole week so kids can see the transition of the stages. (10 minutes)

Demonstrate the life cycle with a poster drawing/teacher’s aide (like Figure 1). (5 minutes) Have students draw the life cycle for each other with a blank sheet of paper too? (2-5 minutes)

Play mosquito tag [round 1](#_ROUND_1:_Mosquito) (mosquito ecology). (20 minutes)

[**Homework #1**](#HOMEWORK )**:** Mosquito life cycle

Tell students to go home and tell their parents about what they’ve learned and show them their homework.

Duration

1 day

Remind students of this throughout other 5 days

*Day 2, Topic 2:* Types of mosquitoes with emphasis on behavior

What students should know

Three major genera of mosquitoes: *Culex, Anopheles, and Aedes*

**Implication**: There are more than just malaria *Anopheles* mosquitoes of concern.

Two types of mosquitoes bite during the night (*Anopheles* and *Culex*) and *Aedes* bites during the day.

**Implication**: different mosquito behaviors mean that different preventive measures are needed. Mosquito nets used for *Anopheles* won’t always work.

Assignments and activities

Discuss and review with a pictorial handouts like (Figures 2 and 3)

Distribute **mosquito poem** and ask them to memorize by Friday

Duration

Very brief: 10 minutes.

*Day 2, Topic 3:* Diseases Transmitted by Mosquitoes

What students should know

The host (human), vector (mosquito), and parasites/viruses are required for disease transmission.

**IMPLICATION:** Mosquito bites make you sick – don’t let them bite you and the best way is to make sure they aren’t breeding in your areas.

Three mosquitoes transmit different diseases:

Malaria – night mosquito *Anopheles* 🡪 mosquito nets work bc they bite at night so net doesn’t protect from all of them.

Elephantiasis/Lymphatic filariasis; Rift Valley 🡪 *Culex*

Dengue; Chikungunya; Yellow Fever 🡪 *Aedes*

**IMPLICATION:** There’s more than malaria so different mosquito behavior and breeding areas are important (tie to Topic 2).

Assignments and activities

Show pictures

Play mosquito tag [round 2](#_ROUND_2:_Mosquito) (with human influence).

Duration

10 minutes for the diseases.

More time as needed for review/revisiting previous topics.

*Day 3, Topic 4:* Mosquito Breeding Areas

What students should know

Some mosquitoes breed in open water but *Aedes* breed in containers

**Implication:** you are capable of making a difference by preventing mosquito breeding in containers.

Two important factors affecting which containers mosquitoes breed in:

1) length of time water is left out (more than 4 days)

2) mosquitoes’ access to the water (covered/uncovered)

**Implication**: don’t let water sit unattended for days

Six important container types/containers that mosquitoes breed in

small domestic container – sanitation

small domestic container – no purpose

bucket – sanitation

tire – no purpose

domestic animal container – animal water

jerry can – laundry

**Implication:** Only focus on these types of containers for intervention.

Assignments and activities

Show examples of the six important container types and place them around the school to do a school activity that involves walking around and looking for them. Make it fun as a ‘container hunt’ and possibly add a few larvae in some of the containers as an example? Together, Kamoni, Mwashee, and the students fill out one of the nyumbani maps that will be homework (below).

[**Homework #2**](#HOMEWORK ): draw a map of the nyumbani highlighting the number and location of the 6 key container types of concern.

Tell them to go home and talk with their parents.

Duration

1 hour

*Day 4, Topic 5:* Mosquito Prevention and Control Measures

What students should know

Ways to prevent mosquito breeding generally

Cover container

Dump water from container

Turn containers over

Poke holes in container

Recycle/re-use container

Ways to prevent mosquito breeding in the six container types

Jerry can (laundry/bathing/etc.)

_Cover it using a nylon or maize cob

Bucket (laundry/bathing/etc.)

_Cover it using a nylon_or plate

Tire__Poke holes in it or fill it with sand

Small domestic containers (Sanitation)__Turn them over or hang them upside down________

Small domestic containers (No purpose) __Collect them for re-use or recycling. Plant flowers in them___

Animal drinking containers__Empty the water daily_________________________________

Assignments and activities

Bring one of the six container types to school to develop a strategy for controlling mosquito breeding

Divide the class into groups (based on the containers they have at their nyumbani) and make a cover or develop a plan for removing the container/risk.

Play mosquito tag [round 2](#_ROUND_2:_Mosquito) (with human influence).

[**Homework #3**](#HOMEWORK ): Container map with action plan

Tell them to go home and talk with their parents

Duration

1 hour

*Day 5, Recap and Post-Test*

Activities

Recite poem

Review all topics 1-4

Give students mosquito detective badges and have them recite the pledge:

**PLEDGE OF COMMITMENT:**

I …… pledge to endure and eradicate mosquitoes

In my school, my home my village and my school

By keeping water covered

Collecting all potential water collecting containers

And telling others about it

Yes I will ooh God help me

For I am a mosquito detective!

Administer post-test

Duration

1 hour

Aedes Intervention Curriculum Appendices

**Mosquito Control Tag**

*Time***:** 30 minutes per round, likely played in one round per day adding complexity each day.

*Materials Needed***:** rubber bands, open field

*Objectives***:**

Understand the mosquito lifecycle and the importance of rain to transform eggs to pupae to adult mosquitoes.

Understand the role of containers that hold water and are not sealed as places where mosquitoes lay eggs.

Understand the role of humans in controlling mosquito breeding.

*How the game is played:*

There is a game master (teacher) who leads the game. The teacher assigns individuals one of five roles. Roles to be assigned include: mosquito, human, container, rain, and unassigned.

*Roles*

Instructor

Rains (water) blue rubber- band

Water containers - yellow rubber bands

Mosquito - red rubber bands

Human - green rubber bands

Un assigned - no rubber bands

An instructor: gives out instructions according to the various characters

*Start basic and add complexity with different rounds:*

-Assume 35 children.

ROUND 1**:** Mosquito lifecycle and ecology

-Game master assigns ~10 people as containers.

-Game master assigns ~5 people as rain.

-Game master assigns ~8 people as mosquitoes.

Decide on the bounds of the playing field.

All players should have a role, either a rubber band that designates they are a mosquito, rain, or container, or no rubber band if unassigned. Mosquitoes should have at least two red rubber bands so they can breed and make more mosquitoes.

Water container players are positioned at opposite sides of the field at the start of the game.

Other players (rain, mosquitoes) start in a line far from the containers.

The game master claps his/her hand to signal the start of the game.

The rain runs to find the containers.

Those that don’t get tagged by water should sit down, which symbolizes being turned over or covered so water doesn’t get in.

Those that fill with water should attach with the character representing water by hooking up of arms.

Only one water can attach with one container.

If the water and container hook up, one and only one mosquito can breed in the containers with water. This is symbolized by hooking arms with the rains, which has already hooked arms with the container. The mosquito must hook arms with the rain container.

Then, the mosquito and the rain and the container move as a group to breed more mosquitoes. They find an unassigned member and attach arms with that person and give him/her a red rubber band. Only the mosquito on the edge and tag additional people to join the group and make more mosquitoes.

The game master claps his/her hand to signal the end of the game.

ROUND 2**:** Mosquito breeding and the human dimension

-Game master assigns ~7 people as containers.

-Game master assigns ~4 people as rain.

-Game master assigns ~6 people as mosquitoes.

-Game master assigns ~4 people as humans.

Decide on the bounds of the playing field.

All players should have a role, either a rubber band that designates they are a mosquito, rain, or container, or no rubber band if unassigned. Mosquitoes should have at least two red rubber bands so they can breed and make more mosquitoes.

Water container players are positioned at opposite sides of the field at the start of the game.

Other players (rain, mosquitoes, and humans) start in a line far from the containers.

The game master claps his/her hand to signal the start of the game.

The rain runs to find the containers. Rain finds the container and ‘fills it’ by hooking arms. Only one rain can hook arms with one container.

At the same time the rain is trying to find containers, humans compete with the rain to get to the containers first and tag them. If the human tags the container then the container is ‘turned upside down’ and the person sits down and is out of the game.

Any containers that were not tagged by rains or that were tagged by humans should sit down, which symbolizes being turned over or covered so water doesn’t get in.

Humans can then touch mosquito players which ‘kills’ them and removes them from the game by having them sit down.

If the water and container hook up, one and only one mosquito can breed in the containers with water. This is symbolized by hooking arms with the rains, which has already hooked arms with the container. The mosquito must hook arms with the rain container.

Then, the mosquito and the rain and the container move as a group to breed more mosquitoes. They find an unassigned member and attach arms with that person and give him/her a red rubber band. Only the mosquito on the edge and tag additional people to join the group and make more mosquitoes.

The game master claps his/her hand to signal the end of the game.

*Points*

The goal of the game according to a mosquito is to make as many mosquitoes as possible. The goal of the game according to rain is to fill containers and then become the biggest team. The goal of the container is to get water and mosquitoes. The container-rain-mosquito groups get a point for each mosquito they breed (or link arms with).

The goal of the game according to the human is to minimize the number of mosquitoes that are bred. They do this by turning the containers upside down before the rain finds them *and* by killing mosquitoes and taking them out of the game. The humans get a point for every container and mosquito they take out of the game.

*Discussion*

After the game, discuss mosquito ecology. Discuss how many mosquitoes there were at the beginning and how many there were at the end. Discuss why that is, including the importance of containers and water for mosquito breeding and the positive impact humans can have. Then discuss the negative role humans could have.

Pre-Post- Test

Name: _____________________________________________________________ Class: _________________ School: ______________________________________ Date: ________________________________________

**SECTION 1: Knowledge and attitudes**

**Instructions: Choose the single best answer or fill in the blank.**

1. **Name** the four stages of the mosquito life cycle and write if it is in water or air.

| **Stage of mosquito life cycle** | **Is it in water or air?** |
| --- | --- |
| 1. |  |
| 2. |  |
| 3. |  |
| 4. |  |

2. How do mosquitoes cause disease?

People getting bitten by adult female mosquitoes

People bathing with water containing larvae and pupae

People drinking larvae and pupae in the water

Don’t know

3. One of the diseases is caused by *Anopheles* mosquitoes, which one is it?

Malaria

Dengue

Chikungunya

Elephantiaisis

4. One of the following diseases is **not** caused by *Aedes* mosquitoes, which one is it?

Malaria

Dengue

Chikungunya

Yellow Fever

5. One of the following is a good way to protect yourself from diseases caused by *Anopheles* mosquitoes, which one is it?

Eating a balanced diet

Covering water containers

Sleeping under a treated mosquito net

Don’t know

6. One of the following is a good way to protect yourself from diseases caused by *Aedes* mosquitoes, which one is it?

Eating a balanced diet

Covering water containers

Sleeping under a treated mosquito net

Don’t know

7. _______________ is caused by drinking the larvae and/or pupae in water.

Fever

Nothing

Cholera

Don’t know

8. Which of the following is **not** a reason why mosquitoes breed most in containers left outside/outdoor?

They collect rain water

They are used less frequently

Mosquitoes breed outside only

Don’t know

9. Why would you cover your laundry water?

Laundry water should be covered

People should not drink laundry water

All water should be covered

Don’t know

10. The following are ways to prevent mosquito breeding, which one is **not**?

Sleeping under a mosquito net

Covering water in containers

Turning over unused containers and keeping them out of the rain

Don’t know

11. What will you do with the following containers to prevent mosquito breeding?

Jerry can

______________________________________________________________________

Bucket

______________________________________________________________________

Tire

___________________________________________________________________

Small domestic containers (Sanitation)

______________________________________________________________

Small domestic containers (No purpose) ______________________________________________________________________

Animal drinking containers

_____________________________________________________________

**SECTION II: Communication**

12. During the **last school term**, did you talk with anyone about what you learned in school?

YES

NO

*If you marked ‘YES’ to the question above, please answer the questions 15-17 below. Otherwise, continue to #18.*

13. Who did you talk with? *Mark all that apply:*

Mother

Father

Sibling(s)

Other **adult(s)** in the family

Other **child(ren)** in the family: cousins or others

Other **adults** *not* in the family such as neighbors

Other **children** *not* in the family: friends in school

Other **children** *not* in the family: friends out of school

14. What did you discuss? *Mark all that apply:*

Issues related to school fees, uniforms, textbooks, or school meetings

Topics you are learning in your classes: Maths/Kiswahili/Science/English/Social Studies

Issues related clubs or after school activities

Issues related to the mosquito curriculum with Julius Kamoni and Luti Mwashee

Other

15. Please describe what you discussed___________________________________________________________

16. During the **last school term**, did anyone **instruct you** to tell your parents something you learned at school?

YES

NO

*If you marked ‘YES’ to the question above, please answer the questions 19-21 below. Otherwise, you are finished.*

17. Please specify *who* **instructed you** to share something with your family

Name of person ________________________

Role of person__________________________

If yes, what did the person ask you to share with your family?

Issues related to school fees, uniforms, textbooks, or school meetings

Topics you are learning in your classes: Maths/Kiswahili/Science/English/Social Studies

Issues related clubs or after school activities

Other

Please describe what you were asked to share

__________________________________________________________________________________

Did you share the information?

YES

NO

| **Grading only: Y/N** | |
| --- | --- |
| 4 stages |  |
| Water line |  |
| # days |  |
| Parent initials |  |

Homework #1: Mosquito Life Cycle

Name: _______________ Class: _____________ School:______________ Date: _______________

**INSTRUCTIONS:**

Draw the 4 stages of the mosquito life cycle.

Indicate where the water is.

Write the number of days the mosquito life cycle lasts.

Show the homework and discuss with your parent/guardian then record initials below

| **DRAW MOSQUITO LIFE CYCLE HERE** |
| --- |

**Initials of the parent/guardian who you discussed this with: ____________**

Homework #1 Grading Guide: Mosquito Life Cycle

Fill in the gray grid:

| **Grading only: Y/N** | |
| --- | --- |
| 4 stages |  |
| Water line |  |
| # days |  |
| Parent initials |  |

**4 stages = Yes** if student wrote egg🡪larvae🡪pupae🡪adult.

**Water line = Yes** if student correctly drew water line encompassing egg, larvae, and pupae but not adult.

**# days = Yes** if student wrote 7-14 days.

**Parent initials = Yes** if student recorded parent initials.

| **Grading only: Y/N** | |
| --- | --- |
| Map |  |
| # containers |  |
| Water |  |
| Larvae/pupae |  |
| Parent initials |  |

Homework #2: Map Of Containers Outside Home

Name: _______________ Class: _____________ School:______________ Date: _______________

**INSTRUCTIONS:**

Fill in the table below. Write the number of each of the six container types you see. Write if you see any mosquito larvae or pupae in the containers.

On the back of this paper, draw a map of the outside of your home and all of the containers where mosquitoes could breed.

Show this to your parent(s) or guardian(s) and record their initials

| **Container types and purposes** | **Number of containers** | **Number of containers with water** | **Do any containers have mosquito larvae or pupae?**  **(Write Yes or no)** |
| --- | --- | --- | --- |
| **1. Small containers used for sanitation**  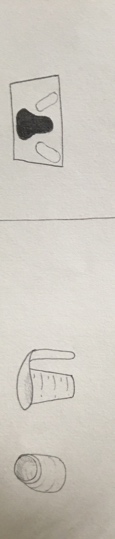 |  |  |  |
| **2. Small containers with no purpose**  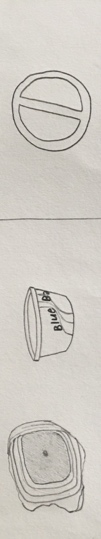 |  |  |  |
| **3. Buckets for sanitation**  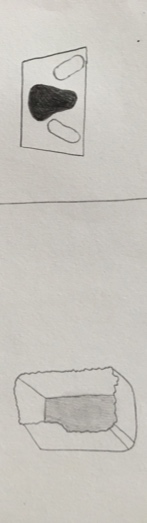 |  |  |  |
| **4. Tire with no purpose**  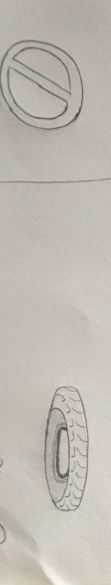 |  |  |  |
| **5. Animal drinking container**  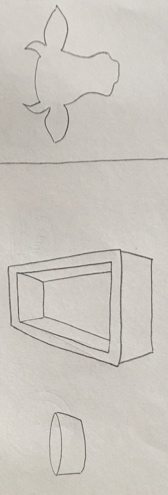 |  |  |  |
| **6. Jerry can or bucket for laundry**  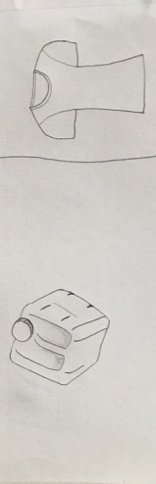 |  |  |  |

**Initials of the parent/guardian who you discussed this with: ____________**

| **DRAW MAP OF HOME AND OUTDOOR CONTAINERS HERE** |
| --- |

Homework #2 Grading Guide: Map of Containers Outside Home

Fill in the gray grid:

| **Grading only: Y/N** | |
| --- | --- |
| Map |  |
| # containers |  |
| Water |  |
| Larvae/pupae |  |
| Parent initials |  |

**Map = Yes** if student properly drew map.

**Number of containers= Yes** if the student properly recorded number of containers.

**Water = Yes** if the student wrote down if water was present.

**Larvae/pupae = Yes** if the student wrote if larvae/pupae were observed

**Parent initials = Yes** if student recorded parent initials

| **Grading only: Y/N** | |
| --- | --- |
| # containers |  |
| Actions described if >0 containers |  |
| Who will take action |  |
| Parent initials |  |

Homework #3: Containers Action Plan

Name: _______________ Class: _____________ School:______________ Date: _______________

**INSTRUCTIONS:**

Fill in the table below. Write the number of each of the six container types you see. If you have 1 or more of the containers at home, write which actions will be taken and who will take the action at home (you, mother, father, sibling, etc.).

Obtain commitment and initials from parents.

| **Container types and purposes** | **Number of containers** | **What actions will be taken?** | **Who will take the action?** |
| --- | --- | --- | --- |
| **1. Small containers used for sanitation**  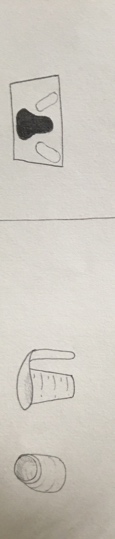 |  |  |  |
| **2. Small containers with no purpose**  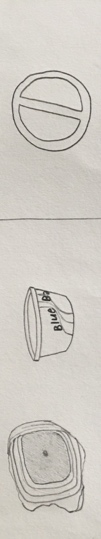 |  |  |  |
| **3. Buckets for sanitation**  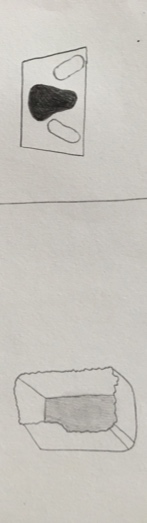 |  |  |  |
| **4. Tire with no purpose**  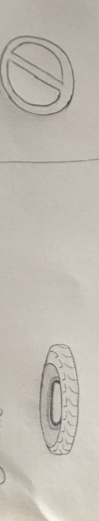 |  |  |  |
| **5. Animal drinking container**  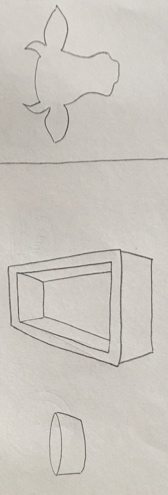 |  |  |  |
| **6. Jerry can or bucket for laundry**  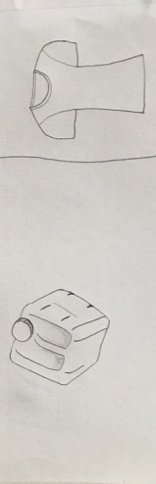 |  |  |  |

**Initials of the parent/guardian who you discussed this with:** _____________

Homework #3 Grading Guide: Container Action Plan

Fill in the gray grid:

| **Grading only: Y/N** | |
| --- | --- |
| # containers |  |
| Actions described if >0 containers |  |
| Who will take action |  |
| Parent initials |  |

Number of containers= Yes if the student recorded number of containers

Actions described if >0 containers = Yes if the student wrote down plausible actions to be taken only if the number of containers was greater than 0.

Who will take action = Yes if the student wrote a family member (or him/herself)

Parent initials = Yes if student recorded parent initials

Mosquito Poem by Luti Mwashee

**MBU**

Mbu ni mdudu hatari,

Wanadamu tujihadhari,

Akituuma anatutia dosari,

Tukijikinga kila kitu shwari.

Magonjwa ni mengi wakitumumunya,

Kama Dengue na Chikungunya,

Yakikupata unakuwa kama panya,

Anavyoliwa na wagunya.

Tufinike maji na ndoo zetu,

Za ndani hadi nje kwetu,

Kama vile wanavyosema wenzetu,

Mbu nje na sisi ndani ya neti zetu.

Tusitupe mikebe ovyo ovyo,

Sote tukinge na mbu kama tulivyo,

Tusijekufa ovyo ovyo,

Nadhani mmenielewa ipasavyo.

Bidii zetu zisipotee bure asilani,

Za kupigana na mbu majumbani,

Mola wape afya Jenna, Joyce, Robin na Kamoni,

Wapigane na mbu leo hadi mwakani.

Beti sita nazifikisha kikomo,

Na sote kwetu ziwe kama somo,

Mwashee narudi kuwa mwanakilimo,

Kwenye mbu tena wala simo.

**MOSQUITOES**

***A mosquito is a dangerous insect,***

***Dear human being lets protect ourselves,***

***When it bites us it affects us,***

***And when we protect ourselves everything is fine.***

***A lot of diseases when they bite us,***

***Like dengue and chikungunya,***

***If you contact them you’ll be like a rat,***

***The way its caught and eaten by the Bajun (a tribe in kenya).***

***Lets cover our water and our buckets,***

***Which are inside and outside our households,***

***Like the way our friends say,***

***Mosquitoes outside and us inside our nets.***

***Lets nots throw containers everywhere,***

***We should all protect ourselves from mosquitoes,***

***So as to avoid deaths,***

***I hope you’ve all understood what am saying.***

***Our hard work should not go fruitless,***

***The way we are fight mosquitoes in the households,***

***God give good health to Jenna, Joyce , Robin and Kamoni,***

***So as to fight mosquitoes today and tomorrow.***

***Am reaching the end of my six stanzas,***

***I hope they are a lesson to all of us,***

***Mwashee am going back to be a farmer,***

***Am leaving alone issues of mosquitoes.***
